# Detection of a highly prevalent and potentially virulent strain of *Pseudomonas aeruginosa *from nosocomial infections in a medical center

**DOI:** 10.1186/1471-2180-5-29

**Published:** 2005-05-20

**Authors:** Ghassan M Matar, Mira H Chaar, George F Araj, Zaher Srour, Ghassan Jamaleddine, Usamah Hadi

**Affiliations:** 1 Departments of Microbiology and Immunology, American University of Beirut, Beirut, Lebanon; 2 Otolaryngology, Neck and Head Surgery, American University of Beirut, Beirut, Lebanon; 3 Pathology and Laboratory Medicine, American University of Beirut, Beirut, Lebanon; 4 Internal Medicine Faculty of Medicine, American University of Beirut, Beirut, Lebanon

## Abstract

**Background:**

We correlated genotypes, virulence factors and antimicrobial susceptibility patterns of nosocomially identified *Pseudomonas aeruginosa *isolates from clinical specimens to those of environmental isolates encountered in the same units of a medical center. Antibiotic susceptibility testing, RAPD analysis and detection of enzymatic activities of extracellular virulence factors, were done on these isolates.

**Results:**

Data showed that most of the clinical and environmental isolates were susceptible to tested antimicrobial agents. RAPD analysis determined the presence of 31 genotypes, with genotype 1 detected in 42% of the clinical isolates and 43% of the environmental isolates. Enzymatic activity testing showed that genotype 1 produced all virulence factors tested for.

**Conclusion:**

In conclusion, our data demonstrated the predominant prevalence of a potentially virulent *P. aeruginosa *genotype, circulating in a number of units of the medical center and emphasize the need to reinforce infection control measures.

## Background

Despite the advances in hospital care and the introduction of a wide variety of antimicrobial agents, *Pseudomonas aeruginosa *continues to be a major nosocomial pathogen particularly in patients who suffer from immunosuppression [1]. *P. aeruginosa *is a ubiquitous pathogen prevalent in the hospital environments, and can cause severe nosocomial infections [2]. The latter involve a broad spectrum of infections including the respiratory, gastrointestinal, and urinary tracts as well as wound infections, sepsis and others [3]. Various possible sources of *P. aeruginosa *infection in hospitals have been identified; such as tap water, medical equipment, hospital personnel and other patients [2,4]. *P. aeruginosa *accounts for 10% of all hospital acquired infections, a site specific prevalence which may vary from one unit to another and from study to study [5]. Among data on site-specific infections, *P. aeruginosa *appears to be the major cause of ventilator-associated pneumonia with a high rate of attributable mortality [6]. Moreover this organism can contaminate a number of other medical equipment such as respirators, endoscopes, bronchoscopes, transvenous pacemakers, urinary catheters, and dialysis equipment, leading to site-related infections [7,8]. During the last year, the average prevalence of *P. aeruginosa *nosocomial infections in our medical center was 18%. Such a high rate prompted us to study the *P. aeruginosa *genotypes circulating in the various units to reveal the clonal relationship between clinical and environmental isolates and to allow elucidating the source and mode of transmission of this important bacterium at this medical center.

## Results

Our data have shown that several mechanical devices were associated with *P. aeruginosa *infection in our patients with 55% due to mechanical ventilation and the remaining 45% due to polysite catheters, Foley catheters and different types of surgery.

**Figure 1:**
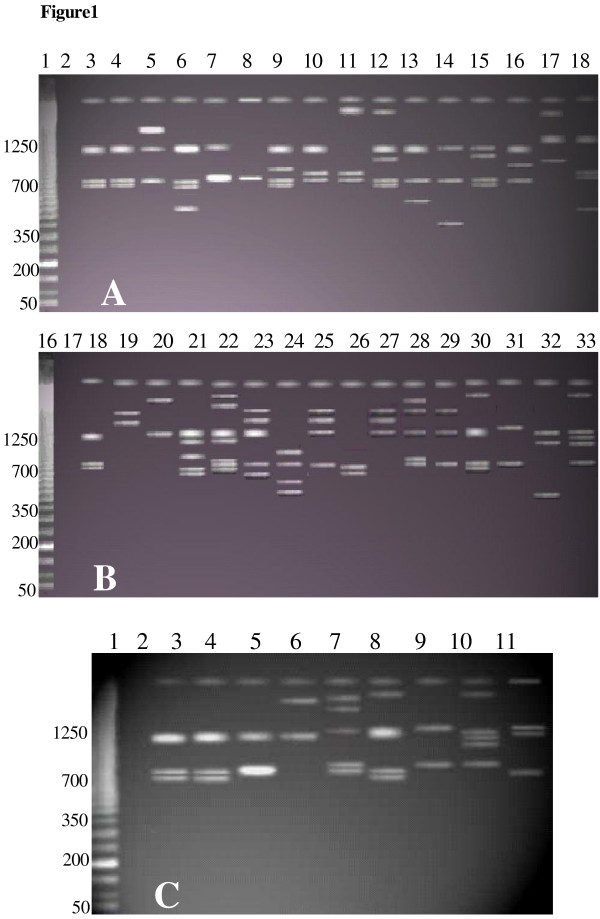
(A) Genotypes of *P. aeruginosa *isolates found in 10 units. Lane 1: 50-bp ladder, lane 2: negative control, lane 3:ATCC25321 strain, lanes 4–18: Genotypes 1,2,3,4,5,6,7,8,9,10,11,12,13,14, and 15. (B) Lane 16: 50-bp ladder, lane 17: negative control, lane 18: ATCC 25321 strain, lanes 19–33: Genotypes 16,17,18,19,20,21,22,23,24,25,26,27,28,29, and 30. (C) Genotypes of *P. aeruginosa *from environmental sources found in the RCU. Lane 1: 50-bp ladder, lane 2: negative control, lane 3:ATCC 25321 strain, lane 4–11: Genotypes (1,4,17,25,27,28,30,31).

**Figure 2:**
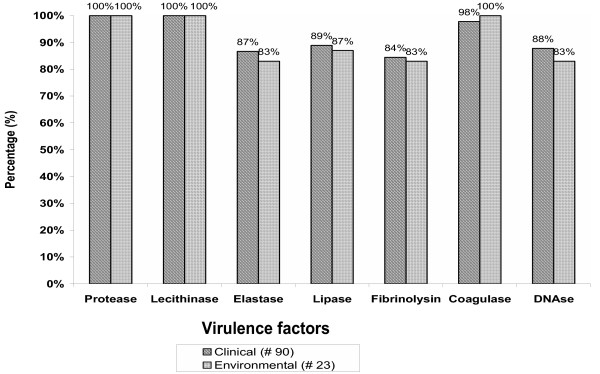
Percentage of clinical and environmental *P. aeruginosa *isolates producing the virulence factors.

RAPD analysis have shown 31 genotypes present among the clinical and the environmental isolates (figure 1). Thirty eight of ninety (42%) of the clinical isolates and 10/23 (43%) of the environmental isolates showed genotype 1 to be distributed among the medical center units. Each of the genotypes 2–30 represented from 1–8% of the strains. Antimicrobial susceptibility testing has shown that susceptibility patterns between clinical and environmental isolates were similar with most drugs showing > 90% susceptibility, with the exception of tobramycin and gentamycin which showed lower susceptibility (83%) in the environmental strains. Data is in concordance with previous reports in this institution [13]. Enzymatic assays determined that genotype 1 in addition to other 10 genotypes, in clinical and environmental isolates, were positive for all virulence factors tested for. All other genotypes were all positive for the production of protease, lecithinase and coagulase and positive in 83–89% of the cases for the production of the other 4 virulence factors (Figure 2). Table 1 shows the relationship between the various genotypes, to source, antibiograms and virulence factors.

## Discussion

The strain distribution based on RAPD analysis showed that clinical and environmental isolates distributed in one or more than one unit, included genotypes 1–8 and 9, with predominance of genotype 1 in all investigated units. This may indicate that cross contamination among patients lead to the spread of this genotype among the various units, possibly through transient hand carriage by health care personnel due to contact with contaminated surfaces or by patient contact with contaminated surfaces or medical equipment [4]. Our findings suggest that cross colonization may be an important means of *P. aeruginosa *spread and infection especially after identification of a potentially virulent clone (genotype 1) of this organism that had been propagated in various units over a period of 9 months. This could indicate that the patients were continuously infected with a strain originating from an exogenous source. The importance of cross colonization of *P. aeruginosa *in nosocomial infections was previously reported, by Bergmans *et al *who studied 100 patients admitted to an ICU ward; cross colonization accounted for 50% of all cases of acquired *P. aeruginosa *colonization, the other 50% of patients were probably colonized from endogenous sources [14].

The remaining genotypes were isolated exclusively from the clinical specimens of patients and were not detected among environmental isolates. Most of these genotypes did not harbor all the virulence factors tested for. Not being encountered in environmental sources may indicate that these strains may have been endogenously acquired. High rate of colonization with *P. aeruginosa *from endogenous sources occurs mainly in the respiratory and gastrointestinal tracts [2]. Hospitalization may lead to increased rates of carriage, particularly in the lower respiratory tract in patients undergoing mechanical ventilation, upper respiratory tract due to broncho- pulmonary colonization and infection, in the gastrointestinal tract of patients receiving chemotherapy for neoplastic diseases, or at virtually any site in patients treated with antibiotics [3,15,16].

Prevalence of strains with resistance to all antimicrobial agents will constitute a major risk for hospitalized patients. In our study, though, the most prevalent strain of genotype 1 was susceptible to all antimicrobial agents and does not constitute a problem in treatment. However, its potential of producing all virulence factors and being spread by various means in the hospital, unlike other genotypes that harbor all virulence factors, may render genotype 1 a high risk pathogen specially in the immunosuppressed and debilitated patients. The fact that genotype 1 remained susceptible to all antibiotics lead to the conclusion that patients could be continuously infected with a strain originating from an exogenous source.

In addition to mechanical spread by personnel, this genotype may carry a number of adhesins that enhance its colonization in the hospital environment and render it more accessible to patients. Studies are underway in our laboratory to detect by PCR, genes encoding a number of adhesins and determine their transcription levels in genotype 1, in comparison to other encountered genotypes. This will shed light on the possible role of adhesins on the prevalence of this genotype in the hospital environment. The extracellular enzymes or toxins produced, on the other hand, will contribute to the breaking down physical barriers and help the organism to penetrate, impair host defenses, and render its new milieu more conducive to its physical, nutritional and reproductive requirements [17].

In summary, our data have shown the predominant prevalence of a potentially virulent *P. aeruginosa *genotype 1 in clinical and environmental specimens, circulating in the various hospital units. From a practical point of view, the results of our study emphasize the need to reinforce implementation of infection control measures, and to limit the transmission of *P. aeruginosa *among patients and from environmental sources to patients. Screening for *P. aeruginosa *carriage in all patients with nosocomial colonization or infection should also be done in research settings using both rectal and respiratory tract specimens to determine the source of colonization/infection and hence get informed on whether it was acquired endogenously or exogenously.

## Conclusion

In conclusion, our data have shown the predominant prevalence of a potentially virulent *P. aeruginosa *genotype 1 in clinical and environmental specimens, circulating in the various medical center units.

## Methods

Consecutive *P. aeruginosa *(90) isolates were recovered from different patients specimens (one per patient) submitted for bacteriological investigations at the Clinical Microbiology Laboratory at the American University of Beirut Medical Center (AUBMC), between September 2003 and May 2004. Patients acquiring a nosocomial infection due to *P. aeruginosa *as determined by clinical and laboratory testing and indicated in their medical records, were only considered in this study. Fever, and recovery of *P. aeruginosa *from the site of infection during stay at the medical center, constituted the most important criteria that defined patient's infection with this organism. Patients' data collected from the medical records, included age (2 to 91 years), sex (Males: 43 and Females: 47), admission date, admission diagnosis, invasive procedures used on the patients, symptoms, and the date of the first positive culture for *P. aeruginosa*. The drugs used in the treatment of these patients, mainly included: amikacin, azactam, gentamicin, tobramycin, and tazocin. Patients were distributed within ten different units in the medical center, mainly the Respiratory Care Unit (RCU), Intensive Care Unit (ICU), Coronary Care Unit (CCU), the Surgery Unit, as well as other units. Twenty three isolates were also collected from different environmental sources, such as, respirators, respirators' filters, water irrigation, tap water, basins, trays, bed side tables, side rails, and sink sides. Statistical analysis determined the sample size required to estimate the true proportion (percentage of *P. aeruginosa *infections during a given period of time) to within 0.10, with 95% confidence was calculated. Calculations estimated the minimum number of samples required to be 54 [9].

The presumptive identification of *P. aeruginosa *on culture based on colonial morphology, Gram stain microscopy, and oxidase test was further confirmed by the API NE Kits and growth at 42°C. Susceptibility of all isolates to a panel of antimicrobial agents (amikacin, aztreonam, ceftazidime, ciprofloxacin, gentamicin, imipenem, piperacillin/tazobactam, tobramycin) was determined according to the guidelines of the National Committee for Clinical Laboratory Standards (NCCLS) [10]. *P. aeruginosa *ATCC 25321 strain was used as positive control in all tests.

DNA was extracted from *P. aeruginosa *ATCC strain and from all isolates of *P. aeruginosa *by the GFX™ Genomic Blood DNA Purification Kit (Amersham PharmaciaBiotech, Uppsala, Sweden) according to the manufacturers' specifications. Random amplified polymorphic DNA (RAPD) analysis of the clinical and environmental isolates using two in-house oligonucleotide primers, Pa1 (5'AGGGGTCTTG 3') and Pa2 (5' CTTCTTCAGCTCGACGCGACG 3') was done. RAPD was carried out according to Matar et al [11] using the PTC-100™ Programmable Thermal Controller (MJ Research, Inc., Watertown, Mass., USA). Briefly, RAPD was carried out on all isolates in 100 μl reaction mixtures containing each: 10 μl of template DNA, 16 μl of dNTPs (0.2 mM), 10 μl of 10X PCR buffer (100 mM TrisHCl [pH 8.3], 500 mM KCl, 4 mM MgCl_2_), 1 μl of primer 1 (0.5 μM), 1 μl of primer 2 (0.3 μg/ μl), 2.5 U of *Taq *DNA polymerase and 61.5 μl of nanopure sterile water. The amplification program, included the following steps: denaturation at 94°C for 3s, annealing at 53°C for 1 min and extension at 72°C for 1 min, for 44 cycles. The cycles were followed by a final extension step at 72°C for 10 min. Amplicons were subjected to electrophoresis on 2% agarose gels at 107 volts for 2 hours. Patterns that had the same number of bands and similar fragments size were considered identical.

The enzymatic activities of the isolates of *P. aeruginosa *were evaluated by spot inoculation containing 10^6 ^CFU/ml of the organisms in various media [12]. Media used: Brain heart infusion for the protease activity, 1% elastin (Sigma Chemical Co. St Louis, Mo. USA) for the elastase activity, human fibrinogen type 1 (Sigma Chemical Co. St Louis, Mo. USA) for the fibrinolytic activity, trypticase soy agar (TSA) supplemented with egg yolk (Difco Laboratories, Detroit, Mi., USA) for the lecithinase production, TSA with tween 80 (Sigma Chemical Co. St Louis, Mo. USA) for the lipase activity, DNase test agar with toluidine blue 0, (Difco Laboratories, Detroit Mi., USA) for the DNAase production, and rabbit plasma (Difco Laboratories, Detroit Mi., USA) for the coagulase activity. Positivity of tests was assessed as follows: clearing of opacity around the inoculum spots for the protease, elastase and fibrinolytic activities, white precipitate around or beneath the inoculum spots for the lecithinase activity, a turbid halo around the inoculum spots for the lipase activity, formation of a pink halo around the inoculum spots for the DNase activity, and gelling of rabbit plasma after 48 hours for the coagulase activity.

## Authors' contributions

GM supervised the study and wrote the manuscript. MC did the bench work and helped in writing the manuscript. GA provided bacterial isolates. ZS provided help on wards. GJ provided clinical support. UH provided clinical support. All authors read and approved the final manuscript.

## Figures and Tables

**Table 1:** Correlation between *P. aeruginosa *genotypes, source and site of infection, antimicrobial susceptibility and enzymatic activities of virulence factors

**Genotypes**	**types of unit**	**Number of clinical isolates**	**Isolation site/ Number of isolates**	**Antibiograms**									**Virulence factors**				
				
				AN	ATM	CAZ	CIP	GM	IPM	TZ	NN	Pro	Lec	Elas	Lip	Fib	Coag
1	RCU	15	DTA^1^/ 13 blood/ 1 Wound/ 1	S	S	S	S	S	S	S	S	+	+	+	+	+	+
	ICU	3	DTA/ 2 Wound/ 1	S	S	S	S	S	S	S	S	+	+	+	+	+	+
	CCU	1	Urine/ 1	S	S	S	S	S	S	S	S	+	+	+	+	+	+
	PICU	4	DTA/2 Tongue/ 1 Wound/ 1	S	S	S	S	S	S	S	S	+	+	+	+	+	+
	St Jude	1	Wound/ 1	S	S	S	S	S	S	S	S	+	+	+	+	+	+
	Surgery	3	Wound/ 3	S	S	S	S	S	S	S	S	+	+	+	+	+	+
	5 South	6	Abscess/ 1 Sputum/ 2 DTA/ 1 Bronchial washing/ 1 Urine/ 1	S	S	S	S	S	S	S	S	+	+	+	+	+	+
	4 South	2	Sputum/ 1 Swab/ 1	S	S	S	S	S	S	S	S	+	+	+	+	+	+
	9 South	3	DTA/ 1 Sputum/ 1 Blood/ 1	S	S	S	S	S	S	S	S	+	+	+	+	+	+
2	RCU	1	DTA/ 1	S	S	S	S	S	S	S	S	+	+	+	+	+	+
	ICU	1	DTA/ 1	S	S	S	S	S	S	S	S	+	+	+	+	+	+
	Surgery	2	Wound/ 2	S	S	S	S	S	S	S	S	+	+	+	+	+	+
	Oncology	1	Blood/ 1	S	S	S	S	S	S	S	S	+	+	+	+	+	+
	5 south	1	Pleural fuild/ 1	S	S	S	S	S	S	S	S	+	+	+	+	+	+
3	RCU	2	DTA/ 1 Wound/ 1	S	S	S	S	S	S	S	S	+	+	+	+	+	+
	5 South	2	Sputum/ 2	S	S	S	S	S	S	S	S	+	+	+	+	+	+
	Oncology	3	DTA/ 2 Blood/ 1	S	S	S	S	S	S	S	S	+	+	+	+	+	+
4	RCU	1	DTA/ 1	S	S	S	S	S	S	S	S	+	+	+	+	+	+
	St Jude	2	Blood/ 1 Catheter/ 1	S	S	S	S	S	S	S	S	+	+	+	+	+	+
	5 South	1	Bile/ 1	S	S	S	S	S	S	S	S	+	+	+	+	+	+
5	PICU	1	DTA/ 1	S	S	S	S	S	S	S	S	+	+	-	+	+	+
	4 South	1	DTA/ 1	S	S	S	S	S	S	S	S	+	+	-	+	+	+
6	RCU	1	DTA/ 1	S	S	S	S	S	S	S	S	+	+	+	-	+	+
	ICU	1	Sinus/ 1	S	S	S	S	S	S	S	S	+	+	+	-	+	+
7	ICU	2	DTA/ 2	S	S	S	S	S	S	S	S	+	+	+	-	+	+
	Oncology	1	Blood/ 1	S	S	S	S	S	S	S	S	+	+	+	-	+	+
8	ICU	1	DTA/ 1	S	S	S	S	S	S	S	S	+	+	+	+	-	+
	Oncology	1	Blood/ 1	S	S	S	S	S	S	S	S	+	+	+	+	-	+
9	RCU	1	Urine/ 1	S	S	S	S	S	S	S	S	+	+	+	+	+	+
	Surgery	1	Wound/ 1	S	S	S	S	S	S	S	S	+	+	+	+	+	+
10	4 South	2	Wound/ 2	S	S	S	**R**	**R**	**R**	**R**	**R**	+	+	-	+	-	+
11	Oncology	2	leg ulcer/ 1 Bronchial washing/ 1	S	S	S	S	S	S	S	S	+	+	+	+	+	+
12	5 South	2	DTA/ 2	S	S	S	S	S	S	S	S	+	+	+	-	-	+
13	9 South	1	Sinus discharge/ 1	S	S	S	**R**	**R**	S	S	**R**	+	+	-	+	-	+
14	9 South	1	Wound/ 1	S	S	S	S	S	S	S	S	+	+	+	+	+	+
15	ICU	1	DTA/ 1	S	S	S	S	S	S	S	S	+	+	+	-	+	+
16	CCU	1	Sternal tissue / 1	S	S	S	S	S	S	S	S	+	+	+	+	+	-
17	CCU	1	Urine/ 1	S	S	S	S	S	S	S	S	+	+	+	-	+	+
18	CCU	1	DTA/ 1	S	**I**	S	S	S	S	S	S	+	+	-	-	-	+
19	RCU	1	DTA/ 1	S	**I**	S	S	S	S	S	S	+	+	+	+	+	+
20	RCU	1	DTA/ 1	S	S	S	S	S	**R**	S	S	+	+	-	+	-	+
21	RCU	1	DTA/ 1	S	S	S	S	**R**	S	S	**R**	+	+	-	+	-	+
22	RCU	1	DTA/ 1	S	**I**	S	S	S	S	S	S	+	+	+	+	+	+
23	RCU	1	DTA/ 1	S	**I**	S	S	S	S	**R**	S	+	+	-	+	-	+
24	RCU	1	Wound/ 1	**R**	S	**R**	**R**	**R**	**R**	**R**	**R**	+	+	-	+	-	+
25	RCU	1	DTA/ 1	S	S	S	S	**R**	S	S	**I**	+	+	+	+	+	+
26	RCU	1	Sputum/ 1	S	**R**	**R**	S	S	**R**	**R**	S	+	+	-	+	-	+
27	PICU	1	DTA/ 1	S	**R**	**R**	S	S	S	**R**	S	+	+	+	+	+	+
28	ICU	1	DTA/ 1	S	**R**	**R**	S	S	**I**	**R**	S	+	+	+	+	+	+
29	4 South	1	DTA/ 1	S	**I**	S	S	**R**	**R**	S	S	+	+	+	+	+	-
30	RCU	1	DTA/ 1	S	S	S	S	**R**	S	S	**I**	+	+	-	+	-	+

NB: Genotype 31 was only found among the environmental isolates

^1 ^DTA: Deep Tracheal Aspirate

AN: Amikacin

CIP: Ciprofloxacin

TZ: Tazocin

ATM: Aztreonam

GM: Gentamicin

NN: Tobramycin

CAZ : Ceftazidime

IPM: Imipenem

Pro: Protease

Lip: Lipase

Lec: Lecithinase

Fib: Fibrinolysin

Elas: Elastase

Coag: Coagulase
